# Supplementary material for: Brindley’s Glands Volatilome of the Predator Zelus renardii Interacting with Xylella Vectors
Source: Insects. 2023 Jun 3;14(6):520. doi: 10.3390/insects14060520 (PMC10299015; doi:10.3390/insects14060520)
Supplement: Supplementary file 1 [file insects-14-00520-s001.zip › insects-2353769-supplementary.pdf]

Supplementary material

Table S1: VOCs produced by *Z. renardii* only. Abbreviations: M-VOCs = major VOCs; m-VOCs = minor VOCs; R.T. = Retention Time; P.H. = Peak Height; N° Rep. = Number of replicates in which the chemical was detected. In bold are the chemicals found in at least half of the replicates.

| ***Zelus renardii*** | | | | |
|---|---|---|---|---|
| **Compound** | **R.T. (min)** | **P.H. (ppm)** | **Match (%)** | **N° Rep.** |
| **M-VOCs** | | | | |
| **2-methyl-propanoic acid** | 15.838 | 1631335 | 82 | 5 |
| 2-methyl-pentanoic acid | 19.333 | 8615617 | 72 | 1 |
| **2-methyl-butanoic acid** | 20.346 | 2016627 | 76 | 4 |
| **m-VOCs** | | | | |
| 2-pentanone | 6.123 | 22245 | 74 | 1 |
| 2-butanol | 6.800 | 29187 | 78 | 1 |
| 3-methyl-butanal | 8.476 | 28567 | 91 | 2 |
| 2-pentanol | 10.095 | 28326 | 83 | 1 |
| **3-methyl-1-butanol** | 12.035 | 50936 | 82 | 4 |
| 1-ethyl-4-methyl-cyclohexane | 17.722 | 44109 | 81 | 1 |
| 3,5-dimethyl-octane | 18.126 | 13186 | 50 | 1 |
| 3,6-dimethyl-octane | 18.484 | 33890 | 90 | 1 |
| propyl-cyclohexane | 18.600 | 41630 | 72 | 2 |
| methoxyacetic acid, 2-ethyl-cyclohexyl ester | 19.739 | 44727 | 50 | 1 |
| 1,1,4-trimethyl-cyclohexane | 19.744 | 40179 | 58 | 1 |
| cis-1-methyl-4-(1-methylethyl)-cyclohexane | 20.224 | 25588 | 72 | 1 |
| 1-nonyl-cycloheptane | 20.227 | 19839 | 59 | 1 |
| cyclooctanemethanol | 20.387 | 25796 | 61 | 2 |
| 1-methyl-3-propyl-cyclohexane | 21.101 | 32210 | 80 | 1 |
| cis-2-oxabicyclo[4.4.0]decane | 21.105 | 20596 | 72 | 1 |
| 3,5-dimethyl-3-heptene | 21.232 | 20442 | 53 | 1 |
| 4-propyl-3-heptene | 21.233 | 22782 | 57 | 2 |
| octacosyl trifluoroacetate | 21.402 | 20228 | 72 | 1 |
| 2,6-dimethyl-nonane | 21.700 | 70108 | 81 | 2 |
| (1-methylpropyl)-cyclohexane | 22.142 | 20244 | 50 | 1 |
| (2-methylpropyl)-cyclopentane | 22.144 | 29617 | 55 | 1 |
| (2-methylpropyl)-cyclohexane | 22.327 | 62063 | 83 | 1 |
| methoxyacetic acid, 2-ethylhexyl ester | 22.994 | 37049 | 72 | 1 |
| 8-methyl-heptadecane | 23.356 | 45083 | 59 | 1 |

| | | | | |
|---|---|---|---|---|
| 3,4-dimethyl-undecane | 23.358 | 22567 | 78 | 1 |
| 1-ethyl-2-methyl-cyclohexane | 24.073 | 17192 | 59 | 1 |
| 2,4,4,6-tetramethyl-hept-2-ene | 24.081 | 17826 | 53 | 1 |
| trans-1,3-dimethyl-cyclohexane | 24.082 | 14294 | 50 | 1 |
| 2-methyl-1-methylene-3-(1-methylethenyl)-cyclopentane | 34.108 | 18235 | 64 | 1 |
| 2,5-bis(1,1-dimethylethyl)phenol | 38.214 | 14754 | 64 | 1 |
| 2-methyl-propanoic acid, 1,3-propanediyl ester | 39.667 | 23619 | 50 | 1 |

Table S2: VOCs produced by *P. spumarius* only. Abbreviations: m-VOCs = minor VOCs; R.T. = Retention Time; P.H. = Peak Height; N° Rep. = Number of replicates in which the chemical was detected. In bold is the chemical found in at least half of the replicates.

| ***Philaenus spumarius*** | | | | |
|---|---|---|---|---|
| **Compound** | **R.T. (min)** | **P.H. (ppm)** | **Match (%)** | **N° Rep.** |
| **m-VOCs** | | | | |
| cyclohexane | 6.004 | 20063 | 90 | 1 |
| 2,3-butanedione | 6.114 | 91014 | 90 | 1 |
| 3-methyl-butanal | 8.482 | 32148 | 93 | 2 |
| dimethyl-disulfide | 11.958 | 20061 | 95 | 3 |
| propyl-cyclohexane | 18.600 | 13245 | 50 | 1 |
| benzeneacetaldehyde | 24.729 | 21050 | 82 | 2 |
| 2-phenoxy-ethanol | 31.247 | 9980 | 89 | 3 |
| **2,4,6-trimethyl-benzaldehyde** | 32.535 | 27425 | 95 | 7 |
| isobornyl acetate | 34.102 | 19715 | 64 | 1 |
| 7,7-dimethyl-2-methylene-bicyclo[2.2.1]heptane | 34.103 | 18591 | 60 | 1 |
| 2-methyl-1-methylene-3-(1-methylethenyl)-cyclopentane | 34.105 | 17908 | 58 | 1 |
| isobornyl formate | 34.106 | 16291 | 69 | 2 |
| hexanedioic acid, bis(2-ethylhexyl) ester | 49.789 | 38055 | 64 | 1 |

Table S3: VOCs produced by the interaction between *Z. renardii* and *P. spumarius*. Abbreviations: m-VOCs = minor VOCs; M-VOCs = major VOCs; R.T. = Retention Time; P.H. = Peak Height; N° Rep. = Number of replicates in which the chemical was detected. In bold are the chemicals found in at least half of the replicates.

| ***Zelus renardii – Philaenus spumarius* interaction** | | | | |
|---|---|---|---|---|
| **Compound** | **R.T. (min)** | **P.H. (ppm)** | **Match (%)** | **N° Rep.** |
| **M-VOCs** | | | | |
| **3-methyl-1-butanol** | 12.656 | 256956 | 87 | 5 |
| **2-methyl-propanoic acid** | 15.262 | 802433 | 95 | 7 |

| | | | | |
|---|---|---|---|---|
| 5-(1-methylethylidene)-1,3-cyclopentadiene | 16.905 | 138665 | 72 | 1 |
| **2-methyl-butanoic acid** | 18.643 | 507820 | 68 | 5 |
| 2-methyl-pentanoic acid | 18.741 | 2106728 | 72 | 1 |
| 1-ethyl-2-methyl-benzene | 20.806 | 115927 | 93 | 2 |
| 4-cyclohexyl-decane | 22.338 | 147675 | 90 | 1 |
| 1,2,4-trimethyl-benzene | 22.832 | 140645 | 94 | 1 |
| 4-ethyl-decane | 22.951 | 217784 | 76 | 2 |
| 1-butyl-2-propyl-cyclopentane | 23.852 | 103884 | 58 | 1 |
| 5-ethyl-1-nonene | 23.854 | 128398 | 56 | 2 |
| 1-(ethenyloxy)-octadecane | 29.014 | 122934 | 80 | 1 |
| **m-VOCs** | | | | |
| 2,3-butanedione | 6.116 | 25207 | 85 | 2 |
| 2-pentanol | 10.884 | 34370 | 83 | 1 |
| 2,4-dimethyl-hexane | 13.082 | 66112 | 80 | 1 |
| 2-(pentyloxy)-ethanol, acetate | 17.365 | 21772 | 50 | 1 |
| 1,3,5,7-cyclooctatetraene | 18.012 | 47404 | 90 | 3 |
| hexanoic acid, cyclohexyl ester | 18.265 | 23572 | 50 | 1 |
| 4,5-dipropyl-octane | 18.841 | 65768 | 50 | 1 |
| 2,5,6-trimethyl-decane | 19.304 | 57766 | 59 | 1 |
| octadecyl-trifluoroacetate | 20.777 | 63657 | 53 | 1 |
| 2-ethyl-1,3-dimethyl-cyclohexane | 21.031 | 50495 | 72 | 1 |
| cis-1-ethyl-2-methyl-cyclohexane | 21.108 | 55471 | 72 | 1 |
| 2-amino-6-methyl-benzoic acid | 21.692 | 21331 | 52 | 2 |
| $\gamma$-terpinene | 23.457 | 87288 | 96 | 2 |
| 2-ethyl-1-hexanol | 23.608 | 22146 | 80 | 2 |
| 1,2,4,5-tetramethyl-benzene | 23.777 | 79533 | 94 | 1 |
| 7-methyl-2-decene | 23.988 | 59328 | 52 | 1 |
| 2,4,4,6-tetramethyl-hept-2-ene | 24.076 | 50924 | 50 | 1 |
| 1-ethyl-2-methyl-cyclohexane | 24.079 | 54621 | 50 | 2 |
| 4-ethyl-1,2-dimethyl-benzene | 24.559 | 84189 | 76 | 2 |
| 4,5-dimethyl-nonane | 24.883 | 24371 | 53 | 1 |
| 1-ethenyl-3-ethyl-benzene | 25.029 | 33284 | 91 | 1 |
| (2-methyl-2-propenyl)-benzene | 25.031 | 34302 | 56 | 1 |
| 3-methyl-eicosane | 26.586 | 32955 | 59 | 1 |
| cis-1,4-dimethyl-cyclooctane | 27.351 | 34427 | 90 | 1 |

| | | | | |
|---|---|---|---|---|
| cyclododecane | 27.351 | 34416 | 93 | 2 |
| 3-ethyl-benzaldehyde | 28.502 | 19743 | 55 | 1 |
| 4-dodecene | 29.016 | 88184 | 55 | 1 |
| decanal | 29.018 | 26787 | 82 | 2 |
| 4-methyl-tetradecane | 30.197 | 78737 | 72 | 2 |
| benzothiazole | 30.466 | 19724 | 93 | 2 |
| **2,4,6-trimethyl-benzaldehyde** | 32.543 | 42232 | 92 | 6 |
| thymol | 32.835 | 98201 | 95 | 1 |
| 6-methyl-1-octene | 32.936 | 21872 | 70 | 1 |
| 2,4,6-trimethyl-benzoic acid, methyl ester | 33.470 | 29379 | 93 | 3 |
| butanoic acid, heptyl ester | 34.705 | 13714 | 59 | 1 |
| cyclotetradecane | 34.758 | 37934 | 95 | 1 |
| 2-(methylamino)-benzoic acid, methyl ester | 35.364 | 20864 | 97 | 1 |
| 3-ethyl-5-(2-ethylbutyl)-octadecane | 36.358 | 52995 | 80 | 2 |

Table S4: List of VOCs detected by *P. spumarius* progressive aggregation. Abbreviations: m-VOCs = minor VOCs; R.T. = Retention Time; P.H. = Peak Height, PS= *P. spumarius*.

| ***Philaenus spumarius* intraspecific interaction** | | | | | | | |
|---|---|---|---|---|---|---|---|
| **Compound** | **R.T. (min)** | **P.H. (ppm)** | **Match (%)** | **1PS** | **2PS** | **5PS** | **10PS** |
| **m-VOCs** | | | | | | | |
| dimethyl-disulfide | 11.970 | 34445 | 97 | | | X | X |
| 3,6,6-trimethyl-bicyclo[3.1.1]hept-2-ene | 18.874 | 20553 | 90 | | | X | |
| 1-ethyl-2-propyl-cyclohexane | 19.747 | 19633 | 50 | X | | | |
| (S)-1-methyl-4-(1-methylethenyl)-cyclohexene | 22.464 | 14935 | 90 | | X | | |
| 1,5-dimethyl-1,5-cyclooctadiene | 22.472 | 22190 | 96 | X | | | |
| 1,2,3,5-tetramethyl-benzene | 22.639 | 15393 | 90 | X | | | |
| (+)-2-bornanone | 27.948 | 24886 | 95 | X | X | | |
| 2,4,6-trimethyl-benzaldehyde | 32.557 | 16781 | 86 | X | X | X | X |
| methyl-eugenol | 34.810 | 48146 | 98 | X | | | |
| 2,5-bis(1,1-dimethylethyl)-phenol | 38.235 | 17342 | 78 | | | X | X |

Table S5: PERMANOVA analysis of the volatilome between *Zelus renardii* alone and *Zelus renardii* interacting with *Philaenus spumarius*

| Factor | Df | Sum Sqs | R2 | F | P.value |
|---|---|---|---|---|---|
| Treatment | 1 | 0.6274 | 0.1669 | 2.4043 | **0.03** |
| Residual | 12 | 3.1311 | 0.833 | | |

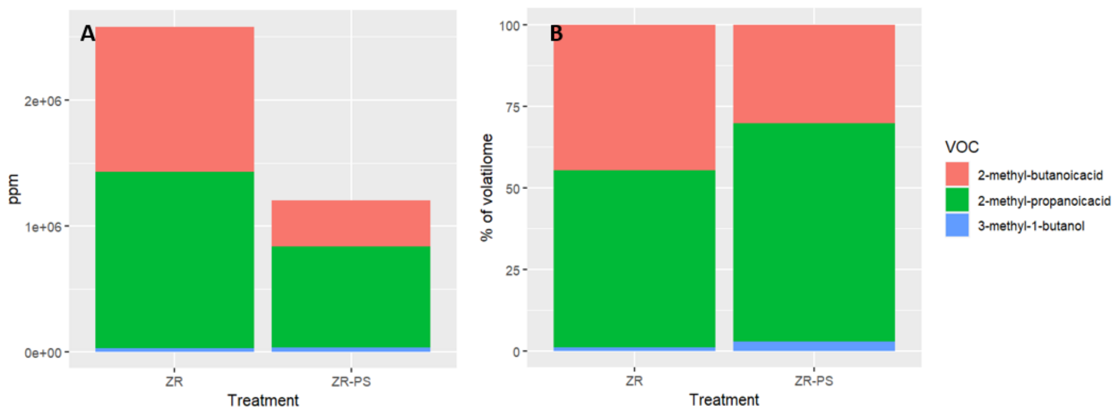


Figure S1: A) parts per million of each VOC of volatilome in each treatment. B) % of each VOC of volatilome in each treatment. Abbreviations: ZR = *Zelus renardii* alone; ZR-PS = *Zelus renardii* interacting with *Philaenus spumarius*.

Table S6: SIMPER analysis showing the volatile that most contributed to the observed differences between groups

| Compound | Contribution | Cumulative contribution | p.value |
|---|---|---|---|
| 2-methyl-propanoic acid | 0.617 | 0.617 | 0.034 |
| 2-methyl-butanoic acid | 0.359 | 0.976 | 0.520 |
| 3-methyl-1-butanol | 0.024 | 1.000 | 0.591 |

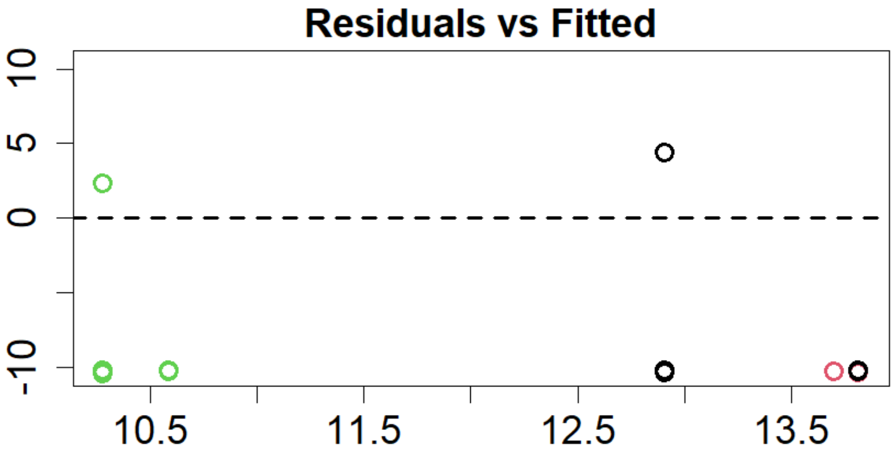


Figure S2: Residual versus fits plot of multivariant glmm with Poisson error distribution

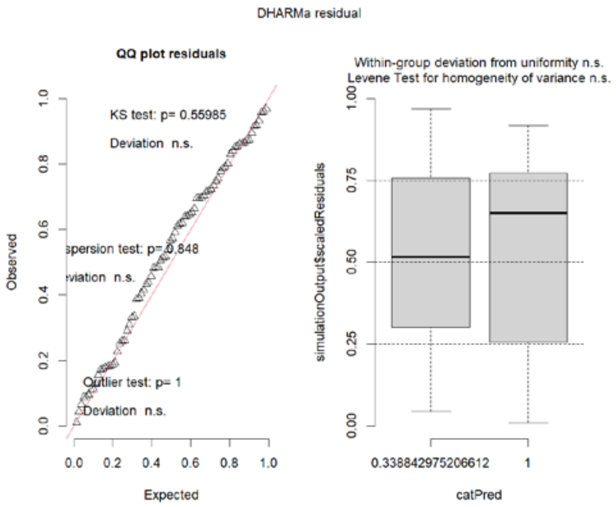


Figure S3: Diagnostic plots of GLMM model (final choice) of 3-methyl-1-butanol acid

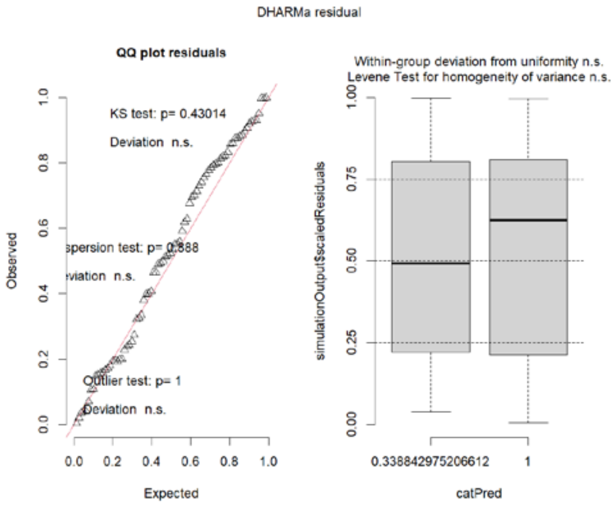


Figure S4: Diagnostic plots of GLMM model (final choice) of 3-methyl-1-butanol acid

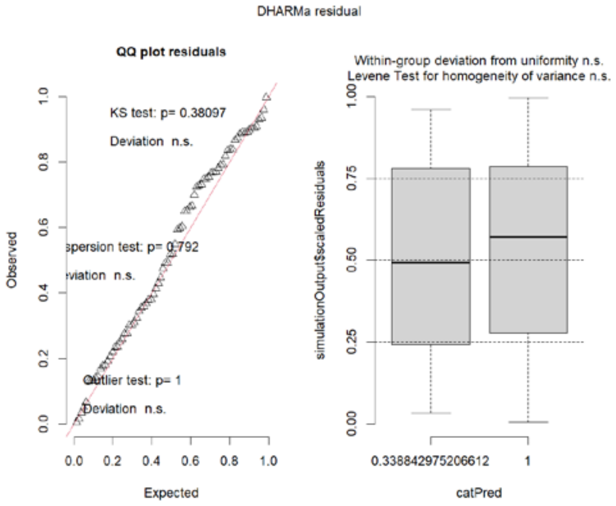

Figure S5: Diagnostic plots of GLMM model (final choice) of 2-methyl-propanoic acid

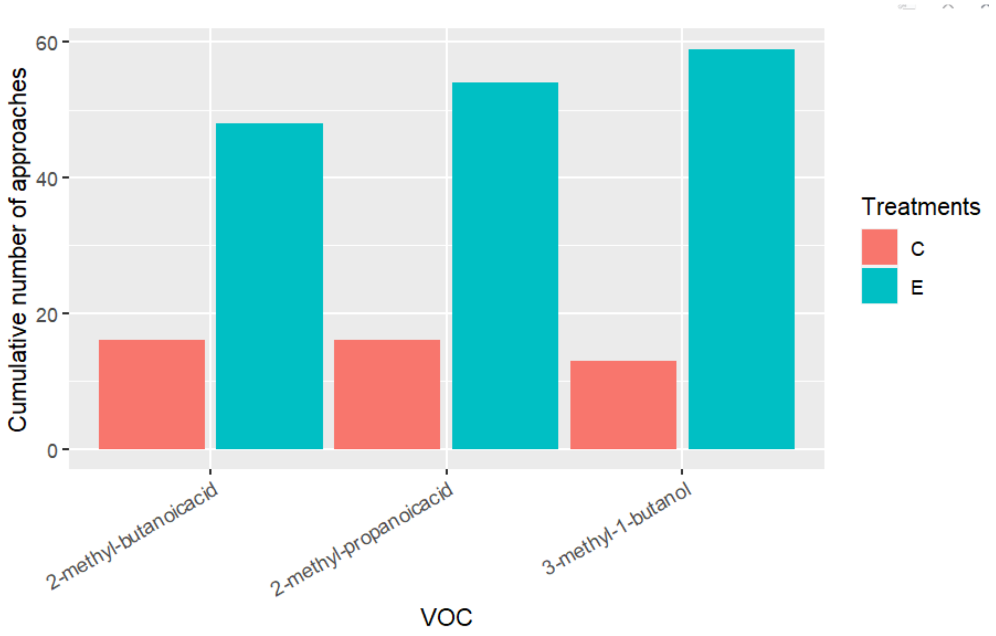


Figure S6: Cumulative number of approaches for each VOC in each treatment. Abbreviations: C = compound; E = empty (control without stimulus)

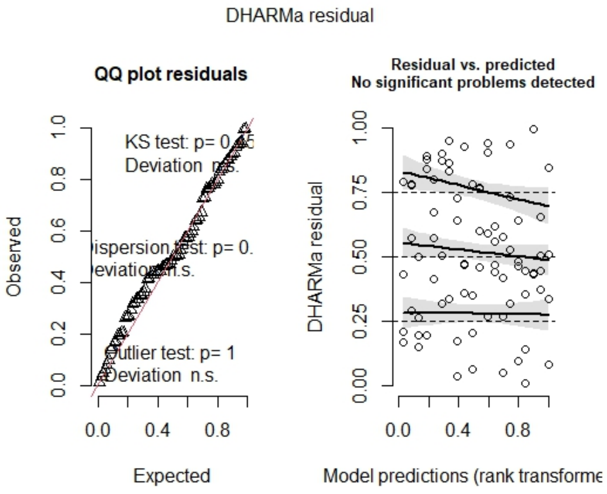


Figure S7: Diagnostic plots of GLMM model (number of approaches) of 2-methyl-propanoic acid

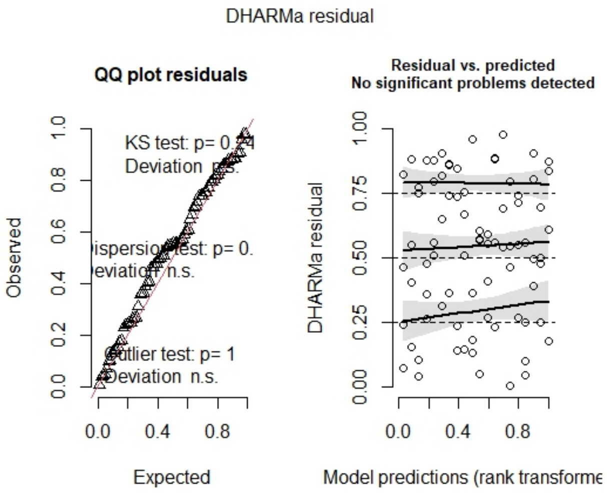


Figure S8: Diagnostic plots of GLMM model (number of approaches) of 2-methyl-butanoic acid

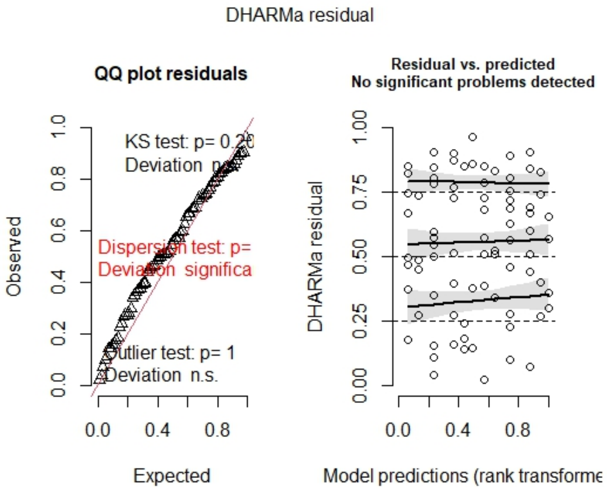


Figure S9: Diagnostic plots of GLMM model (number of approaches) of 3-methyl-1-butanol acid
